# Human Health Risk Assessment and Potentially Harmful Element Contents in the Fruits Cultivated in the Southern Poland

**DOI:** 10.3390/ijerph16245096

**Published:** 2019-12-13

**Authors:** Agnieszka Gruszecka-Kosowska

**Affiliations:** Department of Environmental Protection, Faculty of Geology, Geophysics, and Environmental Protection, AGH University of Science and Technology, Al. Mickiewicza 30, 30-059 Kraków, Poland; agnieszka.gruszecka@agh.edu.pl

**Keywords:** potentially harmful elements, fruits, agricultural soil, human health risk assessment, bioaccumulation index, dietary intake

## Abstract

The presence of potentially harmful elements (PHEs) in popularly consumed fruits in Poland was determined by inductively coupled plasma mass spectrometry. The As, Cd, Co, Cr, Cu, Hg, Ni, Pb, Se, Sb, Tl, and Zn contents were investigated in 21 fruit species grouped as berry, pome, stone, and shell fruits. The PHE contents belonged to the following ranges (mg/kg wet weight): Cd < limit of detection (LOD)–0.116, Co < LOD–0.062, Cu < LOD–15.5, Ni < LOD–2.23, Pb < LOD–2.07, Sb < LOD–0.240, Tl < LOD–0.110, and Zn 0.37–37.7. Their concentrations exceeded the maximum allowable concentration (MAC) set by European Union regulation for Pb only. Bioconcentration coefficient (BC) values, calculated in accordance to the PHE contents in exchangeable and acid soluble forms in soil after first step of the Community Bureau of Reference (BCR) sequential extraction procedure, revealed that berry fruits had potential for accumulation of Cu, Ni, Sb, and Tl; stone fruits—Cu, Sb, and Tl; pome fruits—Cu, Ni, and Sb, and shell fruit (walnut)—Cu. Human health risk assessment associated with the intake of PHEs in fruits was evaluated in terms of daily intake rates (DIR), and carcinogenic and non-carcinogenic risk by cancer risk (CR) and hazard quotient (HQ), respectively. For Pb margin of exposure (MOE) approach was used for health risk evaluation. Daily intake rates for all PHEs were below the provisional maximum tolerable daily intake (PMTDI) values. The mean total non-carcinogenic risk values were the following: berry fruits HQ = 0.47, pome fruits HQ = 0.36, stone fruits HQ = 0.42, and shell fruits (walnut) HQ = 0.22, indicating no health hazards. The carcinogenic risk for As in walnut only under an adult intake scenario (CR = 1.98 × 10^−6^) was found to be above the acceptable risk level. The mean Pb health risk, according to Polish statistical intake rates, was acceptable low as the MOE value was equal to 15.7 for adults. In reference to the intake rates recommended by United States Environmental Protection Agency (USEPA), MOE values for Pb indicated acceptable low risk both for adults (MOE = 14.0) and children (MOE = 1.64). In general, the finding of this research revealed no health risk arising from PHE consumption with fruits for the population of Poland.

## 1. Introduction

Fruits, due to their nutritional attributes [1] and calorific values, became an essential part of a healthy and balanced life style [2]. Food consumption constitutes the primary potentially harmful element (PHE) exposure pathway in humans [3], while soil is the main source of PHEs for edible plants [4]. A high exposure to PHEs results in such negative health effects, as cancers, damage of the nervous system, low birth weight, mental retardation, or damage of the kidney, liver, and other vital organs [5]. In terms of food-chain contamination, attention should be primarily paid to As, Cd, Hg, Pb, and Se [6]. PHE content investigations in edible plants were lately systematically performed globally [4].

The human health risk assessment procedure was developed by the United States Environmental Protection Agency (USEPA) [7] and is currently used globally for determining environmental health hazards’ impact on human health [8]. The health risk implications caused by PHE consumption in fruits are also investigated in various countries lately, especially in these regions, where fruit consumption is relatively high. Research on Pb content in fruits in Algeria [8] and Durban [9] revealed health risk arising from their consumption. In China little potential health risk was also determined for Pb, Cd, Cr, and Ni [10] due to consumption of fruits containing these PHEs. In central Europe (Romania) research on Zn, Cu, Pb, and Cd contents in fruits [11] revealed high potential health risk via their consumption. Moreover, risk values depended strongly on PHE contents in consumed fruits, which depended on the PHE contents in cultivated soil in the first place [11]. Health risk assessment arising from vegetable consumption cultivated on arable soils in Poland revealed that target risk values were exceeded [12], despite the fact that analyzed soils were fully suitable for edible plant cultivation [13]. This suggests that human exposure to environmental threats went beyond the framework of just simple contaminant concentrations in the particular environmental components.

Thus, taking the above into consideration, it is reasonable to hypothesize that the intake of fruits containing PHEs causes a potential risk to human health. Recent research conducted in southern Poland revealed that the total permissible PHE contents were not exceeded in arable soils [13], although the presence of bioavailable PHEs were found in the investigated soils [14]. Therefore, since fresh fruit consumption of locally cultivated fruits is more and more popular currently, it is important to resolve if fruits cultivated on these low PHE contents areas can cause adverse health effects owing to long-term fruit consumption. The goal of the present study was to determine presence of selected PHE concentrations in 21 fruit species popularly consumed in Poland cultivated on recently investigated arable soils [13,14], with the health risk assessment. Detailed objectives of the study included the following: (1) As, Cd, Co, Cr, Cu, Hg, Ni, Pb, Sb, Se, Tl, and Zn contents in berry, pome, stone, and shell groups of fruits, (2) soil-to-plant transfer indices, (3) contribution of PHEs to the daily intake rates via fruit consumption, and (4) assessment of health risk to humans posed by the PHE contents in consumed fruits.

## 2. Materials and Methods

### 2.1. Fruit Sampling and Preparation

**Figure 1:**
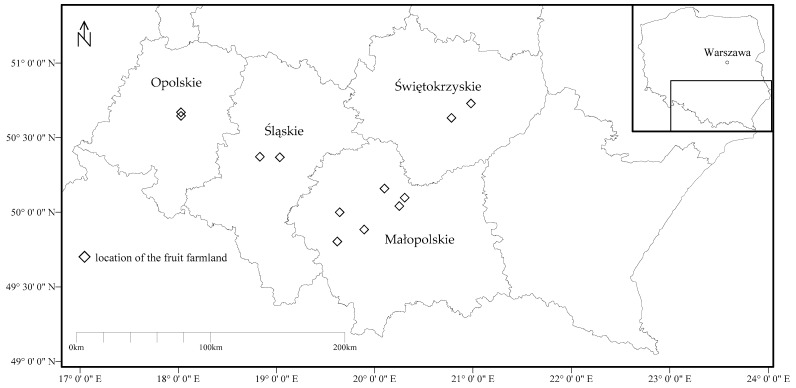
Fruit sampling site locations in the regions of southern Poland (modified from [13]).

The research project investigated PHE content in edible plants cultivated in arable soils with human health risk implications arising from their long-term consumption. Research area located in southern Poland covered four regions, namely the Opolskie, Śląskie, Małopolskie, and Świętokrzyskie (Figure 1). Detailed information on investigated arable soils with total PHE concentration was given in [13] and geochemical fractions of investigated PHEs in soils was given in [14]. Results presented in this paper focused on fruit investigations. Fruit samples were obtained either directly from farmers or bought on the local “fresh food markets” with the reservation that only fruits produced by the local farmers were collected (meaning fruits were not imported from other regions or countries). Not all the fruit species were collected in all investigated regions due to the fact that all typical for Polish agriculture fruits had to be sold locally and had to be cultivated on soils investigated before [13,14]. Thus, during the growing seasons of 2015 and 2016, 21 species of fruits were collected and arranged in four groups: berry fruits (nine species), stone fruits (six species), pome fruits (five species), and shell fruit (walnut only; Table 1). Altogether, 87 fruit samples were analyzed. In case of each sample 1 kg of investigated fruit was collected for further analysis. After transportation to the laboratory, fruit samples were prepared as if for eating: they all were washed and only walnut samples were additionally peeled. The fruits were cut into small pieces, placed on open porcelain dishes and dried under radiant lamps at 70 °C. Dried fruit samples were then ground to obtain coarse powder, using a coffee grinder, and stored in sealed bags for further analysis. The grinder was cleaned after each fruit sample processing to prevent cross-contamination. 

### 2.2. Sample Analyses

Fruit samples (0.5 g, accurate to 0.001 g) were weighed and placed in mineralization flasks, with 15 mL of HNO_3_ and 5 mL of H_2_O_2_ added. Samples were left overnight for organic matter to decompose. The next step involved digestion in the SCP Science DigiPREP HT High Temperature Digestion System at 130 °C during 2 h. After extract solutions were cooled down, their volume was expanded to 50 mL, using ultrapure water. At the same time, blank and reference samples (white cabbage, Certified Reference Material BCR^®^-679) were prepared. The total concentrations of PHEs (As, Cd, Co, Cr, Cu, Hg, Ni, Pb, Sb, Se, Tl, and Zn) were determined by inductively coupled plasma-mass spectrometry ICP-MS (ELAN 6100; Perkin Elmer, Waltham, MA, USA), according to the United States Environmental Protection Agency USEPA 6020B [15] and ISO 17294-2:2003 [16] protocols.

All the results of PHE concentrations in fruits were referred to wet weight (ww.), in accordance to Equation (1). Water content in particular plant samples was taken from the USEPA exposure factors book [17]:

c_ww_ = c_dw_ × (100 − w)/100, (1)

where: c_ww_—concentration of PHE in plant sample, wet weight, c_dw_—concentration of PHE in plant sample, dry weight, and w—percentage water content.

### 2.3. Quality Control

Fruit samples analyses were performed with the observation of the standard certified analytical quality control procedure [18]. To achieve impartial and unequivocal ICP-MS results, elements were also measured, using inductively coupled plasma-optical emission spectroscopy ICP-OES (OPTIMA 7300DV; Perkin Elmer, Waltham, MA, USA), according to the USEPA 6020B [15] and ISO 11885:2009 [19] protocols. The measuring parameters for used ICP spectrometers are given in Table S1. The certified reference material (CRM) (white cabbage, BCR^®^-679) was analyzed at the same time. For the majority of analyzed PHEs, recovery from CRM plant was found to be between 83% and 124%. Method reagent blanks and duplicates were used for quality assurance and quality control purposes. All the reagents used in the laboratory analysis were analytically pure. The results of sample investigations were within the allowable error change values. Analytical bias was statistically insignificant (*p* = 0.05). The precision parameters of ICP-MS and ICP-OES systems were satisfactory, as verified by six different solution injections. For ICP-MS analysis to minimize the impact of interferences, the element correction equations were used for each element. The limit of detection (LOD) values of the investigated PHEs were as follows (µg/dm^3^): As < 0.001, Cd < 0.0005, Co < 0.0005, Cr < 0.0005, Cu < 0.0005, Hg < 0.001, Ni < 0.002, Pb < 0.0005, Sb < 0.0005, Se < 0.002, Tl < 0.001, and Zn < 0.001.

### 2.4. Statistical Analysis

The statistical analysis involved determination of mean, standard deviation, minimum and maximum values, using a Microsoft Excel 2007 spreadsheet. The software package STATISTICA 13 (TIBCO Software Inc., Palo Alto, CA, USA) was used to check data distribution, with the Shapiro-Wilk test (*p* = 0.05). The one-way ANOVA at the 95% confidence level was used to check for any significant differences (*p* ≤ 0.05) between the average PHE concentrations among analyzed groups of fruits. If differences were found to be significant post-hoc analysis were performed followed by the Fisher’s least significant difference (LSD) test. The hierarchical cluster analysis (HCA) and the principal component analysis (PCA) were performed for multivariate statistical modeling of the input data. Halves of the limit of detection (LOD) values were assigned to undetected results in all performed statistical analyses, as recommended by the World Health Organization (WHO) [20].

### 2.5. Soil-to-Plant Transfer Indices

To determine the PHE translocation in the soil-and-plant system, two soil-to-plant transfer factors were used in the present research in order to determine the PHE environmental bioavailability [4]. The bioaccumulation coefficient (BA) described the transference of PHEs from soil to the plant, while the bioconcentration coefficient (BC) described the plant capacity to adsorb PHEs from soil when PHEs occurred in an available form [21]. The BA and BC values were calculated for the investigated groups of fruits, respectively, from general Equations (2) and (3), according to [22]:

BA = C_fruit_/C_st_, (2)

BC = C_fruit_/C_sa_, (3)

where C_fruit_ is the mean concentration of a particular PHE (mg/kg ww.) in the investigated fruit; C_st_ is the mean total concentration of a particular PHE, determined in the soil samples (mg/kg dry weight) from southern Poland, using aqua regia digestion [13]; and C_sa_ is the mean available concentration of a particular PHE, determined in the soil samples (mg/kg dw.) from southern Poland after extraction with (i) 0.11 M CH_3_COOH (the first fraction of the BCR sequential extraction procedure F1) and (ii) 0.05 mol/dm^3^ Na_2_EDTA [14].

### 2.6. Human Health Risk Assessment

The point estimate method developed by United States Environmental Protection Agency (USEPA) [7] was applied to assess the Human Health Risk Assessment (HHRA) arising from the consumption of PHEs in fruits cultivated in southern Poland. The following parameters referring to both adults and children, using the mean and the 95th percentile (P95) of the PHE concentration in fruits, were calculated: the daily intake rate (DIR) values for particular PHEs were calculated as the sum of consumed fruits, according to Equation (4) [23]:

DIR = Σ (C_fruit_ × IR_fruit_/BW), (4)

where C_fruit_ is the concentration of a particular PHE in the group of fruits (mg/kg ww.); IR is the fruit intake rate (g ww./person-day) in the group of fruits; BW is the body weight: 70 kg for adults and 15 kg for children [17].

The intake rates of fruits assumed in this study are presented in Table 2. Three exposure scenarios of fruit consumption were analyzed. The first scenario was designed for adults, based on the available Polish statistical data [24]. The second scenario was based on the fruit intake rates recommended by USEPA [25], since the data on the consumption of certain fruits were missing in the Polish statistics. The third scenario was based on the intake values recommended by USEPA for children, since the Polish statistical data did not involve the subpopulation of children.

According to the statistical data available for adult Poles [24], it was observed that the daily consumption of fruits amounted to 68.3 g ww./person-day. When analyzing the data provided by USEPA [25] in reference to the recommended values for the HHRA calculation in the context of food consumption, it was observed that fruit consumption by adult was equal 100.7 g ww./person-day. In the case of shell fruits (i.e., nuts), the recommended value of daily consumption was used [26,27] since such data were missing in the USEPA guidebook. According to the USEPA data, fruit consumption by children (0–6 years) amounted to 67.4 g ww./person-day; however, the data did not include shell fruit consumption (i.e., nuts) and such information was not available (Table 2).

The average daily doses (ADD) of the PHE ingestion via consumed fruits (mg/kg bw-day) were calculated as the sum of the consumed fruits, using Equation (5):

ADD = Σ (C_fruit_ × IR_fruit_ × EF × ED × 10^−3^)/AT × BW, (5)

where C_fruit_ is the concentration of each PHE in the investigated fruits (mg/kg ww.); IR_fruit_ is the intake rate of fruits (g ww./person-day); EF is the exposure frequency: 365 days/year; ED is the exposure duration: 30 years for adults and 6 years for children [28]; AT is the averaging time in days: ED × 365 for non-carcinogens and 70 years × 365 for carcinogens [28]; and BW is the body weight (kg), as in Equation (4); and 10^−3^ is the unit conversion factor.

The non-carcinogenic PHE risk values from dietary exposure were calculated with Equation (6):

HQ = ADD/RfD,(6)

where HQ is the hazard quotient and RfD is the reference dose for a particular PHE.

The RfD values were set to be as follows (mg/kg bw-day): As 3.00 × 10^−4^, Cd 1.00 × 10^−3^, Co 3.00 × 10^−4^, Cu 4.00 × 10^−2^, Hg 3.00 × 10^−4^, Ni 2.00 × 10^−2^, Sb 4.00 × 10^−4^, Tl 1.00 × 10^−5^, and Zn 3.00 × 10^−1^ [29].

The total non-carcinogenic risk (HQt) value for the investigated PHEs was calculated, using Equation (7):

HQt = HQ_1_ + HQ_2_ + … + HQ_n_, (7)

where HQs are the hazard quotient values for 1–n PHEs investigated in the study.

The carcinogenic risk values of PHEs from dietary exposure were calculated, using Equation (8):

CR = ADD × SF_o_, (8)

where CR is the carcinogenic risk and SF_o_ is the oral slope factor for a particular PHE. 

Only As was considered as a carcinogenic PHE in this study. No SF values were available for other investigated elements at the time. The SF_o_ for As was set to be equal to 1.5 (mg/kg bw-day) ^−1^ [29]. The total carcinogenic risk value, as the sum of partial CR values, was not calculated since As was the only carcinogenic PHE considered in this study.

The Pb risk of dietary exposure was calculated according to the margin of exposure (MOE) approach, as recommended by European Food Safety Authority (EFSA) [30], using Equation (9) [31]:

MOE = BMDL/DIR,(9)

where MOE is the margin of exposure value; BMDL is the benchmark dose (lower confidence limit), estimated at 1.2 µg/kg bw-day for adults and 0.6 µg/kg bw-day [32]; and DIR is the total amount of fruits consumed daily under the analyzed intake scenarios.

## 3. Results and Discussion

### 3.1. PHE Levels in Groups of Fruits

According to the author’s best knowledge the investigations on all available fruit species cultivated on arable soils in Poland were not performed before. Previous studies focused on selected edible plant species or contaminated soil, i.e., [33,34]. Current research characterized contents of twelve PHEs in 21 fruit species typically cultivated in southern Poland in previously investigated soils [13,14]. In the 21 investigated species of fruits, the concentrations of Cr, Hg, Se, and As were <limit of detection (LOD), except for As in walnuts. Consequently, Cr, Hg, and Se were excluded from further analysis. Moreover, bioaccumulation and risk assessment of As in further investigations included only walnut as the sole example of shell fruit. The detectable rates in fruit samples of the remaining PHEs analyzed in this research were the following: Cd 57.5%, Co 22.5%, Cu 97.5%, Ni 23.8%, Pb 58.8%, Sb 45.0%, Tl 30.0%, and Zn 100%. The concentrations of the remaining investigated PHEs, determined in the investigated fruits altogether, were found to belong to the following ranges (mg/kg ww.): Cd < LOD–0.116, Co < LOD–0.062, Cu < LOD–15.5, Ni < LOD–2.23, Pb < LOD–2.07, Sb < LOD–0.240, Tl < LOD–0.110, and Zn 0.37–37.7. In the groups of fruits, the mean PHE concentrations were as follows (in mg/kg ww.): berry fruits: Cd 0.013, Co 0.001, Cu 0.674, Ni 0.218, Pb 0.117, Sb 0.007, Tl 0.004, and Zn 2.55; pome fruits: Cd 0.013, Co 0.006, Cu 0.791, Ni 0.395, Pb 0.042, Sb 0.041, Tl 0.0001, and Zn 2.79; stone fruits: Cd 0.008, Co 0.00003, Cu 0.797, Ni 0.063, Pb 0.169, Sb 0.024, Tl 0.004, and Zn 1.54; and shell fruit (walnut): As 0.039, Cd 0.00003, Co 0.023, Cu 8.105, Ni 0.0001, Pb 0.00003, Sb 0.024, Tl 0.002, and Zn 35.6 (Table 3). The results revealed that the decreasing order of PHE contents was as follows in the groups of fruits: berry fruits Zn > Cu > Ni > Pb > Cd > Sb > Tl > Co, pome fruits: Zn > Cu > Ni > Pb > Sb > Cd > Co > Tl, stone fruits: Zn > Cu > Pb > Ni > Sb > Cd > Tl > Co, and shell fruit (walnut): Zn > Cu > As > Sb > Co > Tl > Ni > Cd > Pb.

The results of the one-way ANOVA test revealed significant differences between the average concentrations of Co, Cu, and Zn in groups of investigated fruits (Table S2). Cobalt and cadmium concentrations were significantly different in shell fruits than in berry, stone, and pome fruits. Zinc concentrations were significantly different in shell fruits than in berry fruits and in stone fruits. Considering investigated regions significant differences between the average concentrations were revealed for Co and Sb in the one-way ANOVA analysis (Table S3). Cobalt concentration was significantly different in Małopolskie than in Opolskie and Śląskie regions. Antimony concentrations were significantly different in Opolskie than in Śląskie and Świętokrzyskie regions.

The principal component analysis (PCA) was performed on the PHE contents in 87 analyzed fruits samples. Results revealed four principal components (PCs), with eigenvalues higher than 1, which altogether explained 88.6% of the variance observed (Table 4). The first principal component (PC1), which accounted for 33.5% of the variance, had high positive loading values (>0.7) for Cu, Zn, and Co. That high positive correlation with the three PHEs corresponded to the highest uptake of essential elements, i.e., Cu and Zn [35] and beneficial elements, i.e., Co [36], as compared to other PHEs. The second principal component (PC2) explained 25.6% of the variance and had high negative loading values of Cd and Ni. That could be correlated with the total concentration of PHEs in soil, as in the cases of Cd and Ni where the negative correlation might indicate that bioavailable element concentration in soils determined element uptake and accumulation by plants [14,37,38]. Moreover, Cd and Ni are characterized by easy bioaccumulation from soil [37,39,40,41,42].

**Figure 2:**
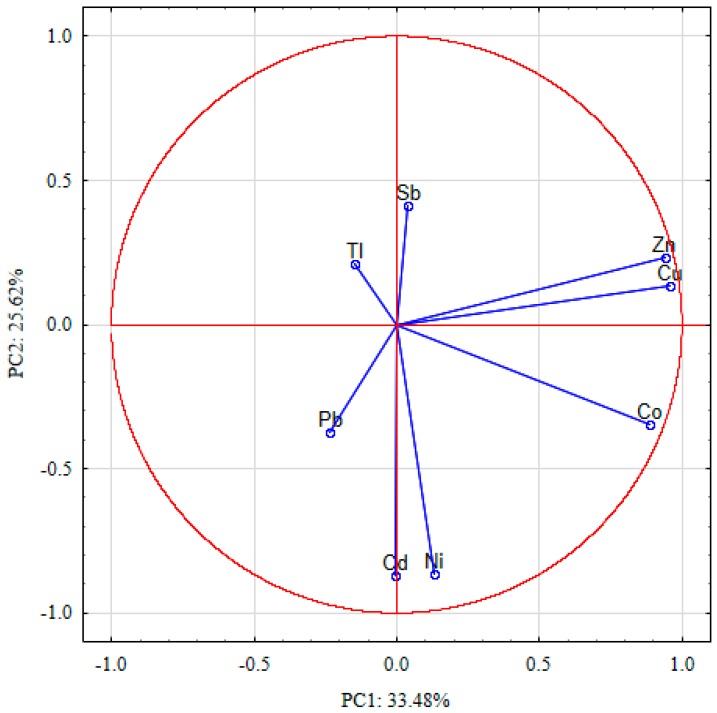
Ordination diagram of PCA, computed for the PHE contents in fruit samples.

The ordination diagram of PCA, computed for the PHE contents in fruits of the first two PC components, was presented in Figure 2. The third component (PC3) explained 16.6% of the variance and had a high positive loading value for Tl and a high negative loading value for Sb. That might be correlated with the plant ability to uptake PHEs from various sources. In the case of Sb, the uptake by plants might be mainly geogenic, or dependent on the element concentration in soil [14], as the function of concentration in parent rocks and non-ferrous ores occurring on the investigated area [43] where particular plants were grown [44,45]. In the case of Tl, uptake by plants might be related to the PHE presence in the atmospheric deposition, generated by anthropogenic activities [46,47]. In the case of the remaining PHEs, their uptake by plants might come from both sources: the effect of the natural occurrence in soil and of the anthropogenic activities related to Zn-Pb [48] and Cu [49] ore mining and processing. The fourth principal component (PC4) explained 12.9% of the variance and had a high negative loading value of Pb. It might be correlated with the ability of plants to transport Pb into aerial parts. Only a limited amount of Pb is transported from roots to higher plant parts [45,50]. The hierarchical cluster analysis (HCA) explored similarities between the analyzed PHE contents in the investigated fruits.

**Figure 3:**
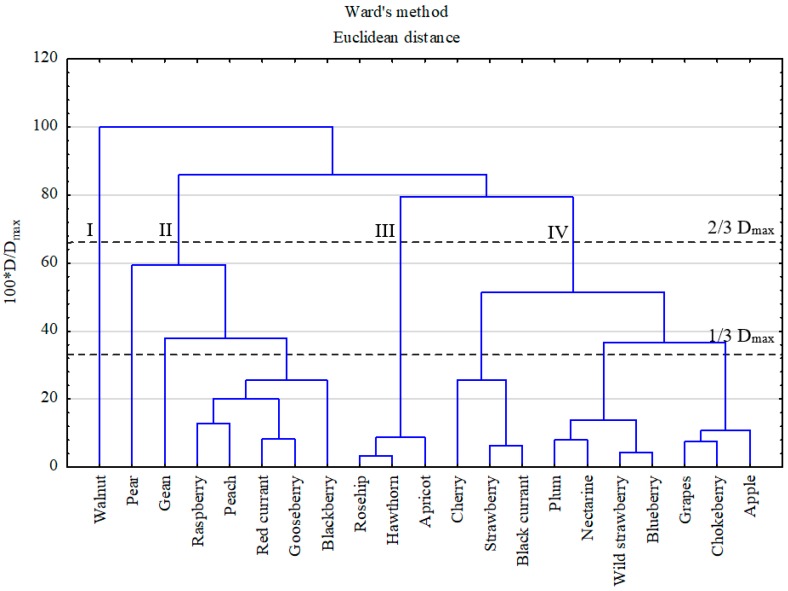
Dendrogram of PHEs in fruits, according to Sneath’s criteria. D: distance, D_max_: maximum distance.

**Figure 4:**
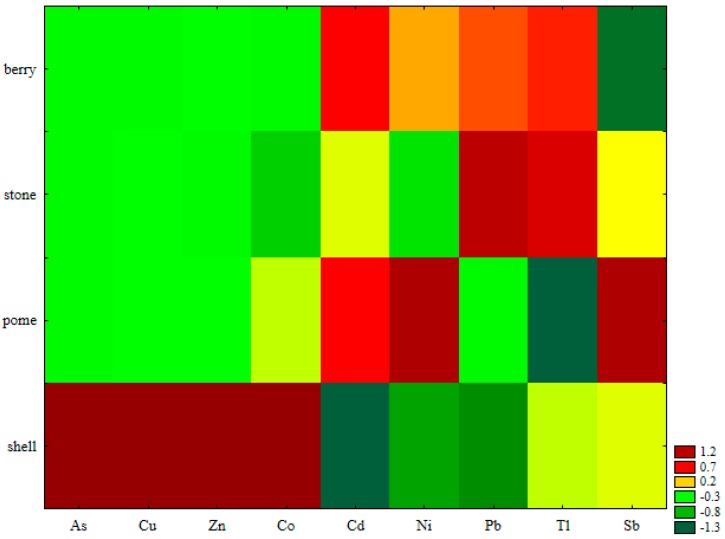
The color-scale map of standardized contents of PHEs in fruit groups.

A hierarchical dendrogram distinguished four similarity groups of variables, according to the Sneath’s criterion equal to 2/3 of the maximum distance (D_max_) (Figure 3). The first and second clusters could be associated with fruits containing the highest concentrations of Cu and Zn, and they are known as plants being rich in those microelements [1,51]. However, the concentrations of non-essential elements were also quite high, i.e., those of Ni and Pb. The first group was separated in respect of the walnut, which as shell fruit, since it contained one order of magnitude more of PHEs, especially of Zn and Cu [52], as compared Group II. The third and fourth clusters could be associated with the fruits containing relatively low concentrations of essential elements (i.e., Zn and Cu), but, at the same time, low concentrations of non-essential elements in Cluster III and high contents of toxic elements in Cluster IV. For a better visualization, standardized PHE contents in specific group of fruits were also presented on a color scale map (Figure 4). The map revealed that the walnut was abundant in Cu, Zn, Co, and As (the only investigated fruit for which the As contents were >LOD). The highest abundance of Ni, Sb, and Cd was observed in pome fruits; that of Pb, Tl, Cd, and Sb in stone fruits, and that of Cd, Pb, Tl, and Ni in berry fruits.

Those results were consistent with the results obtained under other research projects. Cindrić et al. [52] indicated the mean As concentrations in walnuts, sampled in Europe, at the level of 0.027 mg/kg. The results concerning Cu and Zn, obtained in the present study, were about one order of magnitude higher than the contents of Cu and Zn in berry fruits, collected in northern Poland [53], in which region no mineral deposits occur. The contents of Cd in berry fruits samples of the Opolskie region were consistent with the research results performed in the same region [54] as in the present studies. However, the contents of Cd in berry fruits samples were lower in this study than in the fruit samples collected in the Upper Orava region, Slovakia [55], located 20 km south from the border with southern Poland. The contents of Cd, Cu, Pb, Ni, and Zn in pome and berry fruits samples, collected in the Świętokrzyskie region, were consistent with the research results performed on the area east of the Świętokrzyskie region (still in southern Poland) [56,57].

In order to determine the analyzed fruits safety, the PHE content values were compared to the permissible levels stated in the EU regulation on the maximum levels of certain contaminants in foodstuff [58]. The maximum allowable concentration (MAC) values were specified only for two analyzed PHEs (mg/kg ww.): Pb 0.10 (berries and small fruits 0.20) and Cd 0.05. Comparing contents of these elements with permissible levels it was observed that the Pb content exceeded the MAC value in the cases of berry fruits (namely, blackberry, gooseberry, and redcurrant), pome fruit (pear), and stone fruit (peach) and it was on the border of the MAC value in the case of apricot (stone fruit). The Cd contents were on the border of the MAC values only in the case of pear (pome fruit) where the mean Cd value was equal to 0.049 mg/kg ww., while in other fruits Cd contents were much lower than the MAC value for cadmium. The elevated concentrations of Cd and Pb in investigated fruits might be caused due to their significant concentrations in agricultural soils in both total [13] and bioavailable contents [14] for plant uptake. Moreover, it can be also caused by the presence of heavy metals (i.e., Hg, Cd, and Pb) in mineral fertilizers [59] and organic manure [60] commonly used in Poland [61]. Despite the fact that permissible levels of heavy metal contents in investigated fertilizers were not exceeded [59], their commonly usage in the agriculture [62] might cause the elevated content of PHEs in edible plants as well.

### 3.2. Soil-to-Plant Transfer Indices

**Figure 5:**
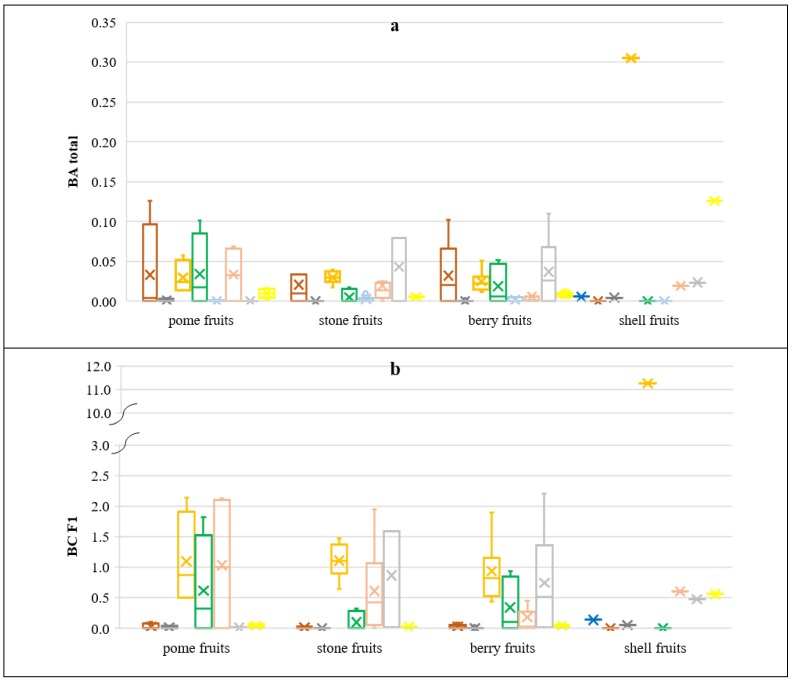

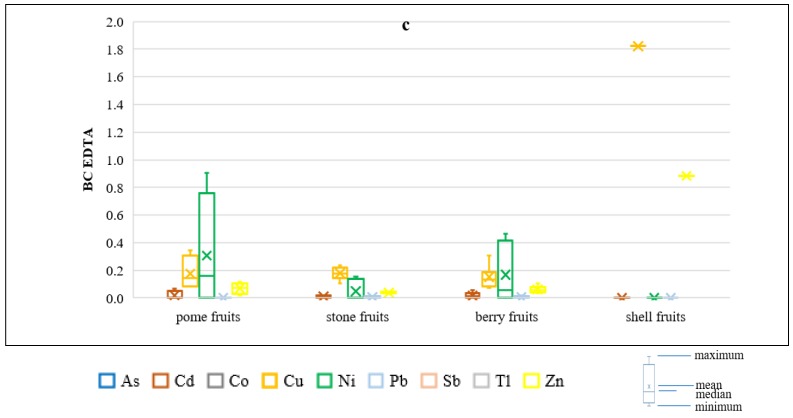
Soil-to-plant transfer indices for the investigated groups of fruits: (**a**) BA_total_—bioaccumulation coefficient, total PHE content, (**b**) BC_F1_—bioconcentration coefficient, PHE content of the F1 fraction of the BCR sequential extraction, and (**c**) BC_EDTA_—bioconcentration coefficient, PHE content: 0.05 mol/dm^3^ of the Na_2_EDTA extraction.

To determine the efficiency of PHE uptake by the investigated fruits BA and BC coefficients were calculated since soil is the main source of elements for plants. Numerical data of the distribution of calculated transfer index values are presented on the box and whisker chart in Figure 5. The bioconcentration coefficient (BC_F1_) values were not calculated for Hg and Pb, since the contents of those PHEs in soil samples were found to be <LOD after the first step of the BCR extraction procedure (exchangeable and acid soluble forms) [13]. Besides, the bioconcentration coefficient (BC_EDTA_) values were not calculated for As, Co, Hg, Sb, and Tl, since those PHEs had not been determined in soil samples after the 0.05 mol/dm^3^ Na_2_EDTA extraction procedure [14]. Contents of PHEs in soils used for bioaccumulation indices calculations are collected based on results obtained in the previous research [13,14] and given combined in Table S4. Moreover, the bioaccumulation coefficient (BA_total_) values were not calculated for Cr, Hg, and Se because 100% of the fruit samples indicated the PHE concentrations of <LOD. All calculated BA_total_ values were <1 (Figure 5a), indicating that none of the investigated fruits had potential for accumulating PHEs, taking into account the total element content in soil. Calculated BC_EDTA_ values (with the exception of Cu in walnut) were also <1 (Figure 5c), which indicated that the investigated fruits had no potential of PHE accumulation when considering the available proportion of PHEs in soils extracted with the 0.05 mol/dm^3^ Na_2_EDTA. However, the analyzes of the BC_F1_ values calculated, with the use of available PHE proportion in soils, extracted with 0.11 M CH_3_COOH (the first fraction of the BCR sequential extraction procedure F1), revealed that the calculated mean BC_F1_ values were >1 (Figure 5b), which pointed the potential for accumulation of Cu in shell fruit (walnut), pome fruits, berry fruits, and stone fruits; Ni in pome fruits and berry fruits; Sb in pome fruits, stone fruits, and berry fruits; and Tl in stone fruits and berry fruits. The investigated groups of fruits were characterized by a similar level of the transfer factor. The highest accumulation potential for Cu and Zn in walnuts was disclosed by Cindrić et al. [52], since walnuts are known to be functional food and an important contributor in supply of essential elements to humans. Other fruits did not reveal any potential for accumulation because plants protect underground parts against the PHE accumulation [63,64]. While plants developed a biochemical mechanism for the PHE adaptation and tolerance in growth media, plant response might vary in various plant species and that should be investigated in particular soil-plant systems [39].

### 3.3. Human Health Risk Assessment

As it was stated above, in the case of Pb and Cd their contents in some investigated fruits exceeded MAC values or were on the border of MAC values, respectively. Moreover, for other investigated PHEs such safe levels were not determined according to the EU regulation. Thus, the HHRA calculations constituted an important area of the presented research.

#### 3.3.1. Daily Intake Rates

**Figure 6:**
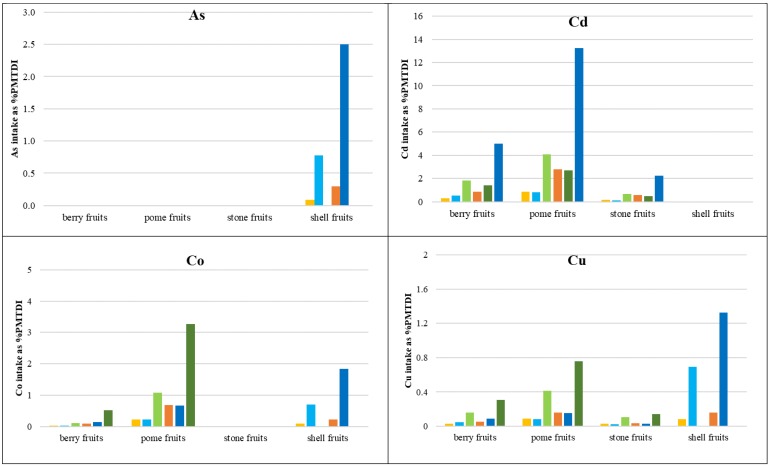

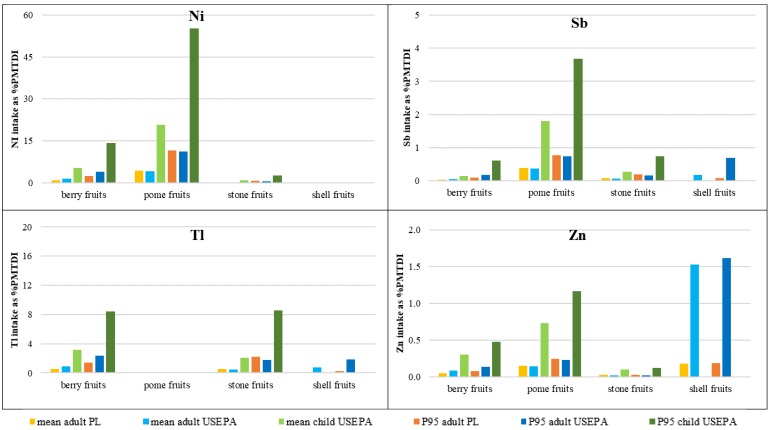
Daily intake rates of PHEs, with consumed fruits, as a % of provisional maximum tolerable daily intake (%PMTDI). Mean adult PL—daily intake rate for adults, statistical data for Poland, mean PHEs concentration; mean adult USEPA—daily intake rate for adults, USEPA recommended values, mean PHEs concentration; mean child USEPA—daily intake rate for children, USEPA recommended values, mean PHEs concentration; P95 adult PL—daily intake rate for adults, statistical data for Poland, the 95th percentile of PHE concentration; P95 adult USEPA—daily intake rate for adults, USEPA recommended values, the 95th percentile of PHE concentration; P95 child USEPA—daily intake rate for children, USEPA recommended values, the 95th percentile of PHE concentration.

The calculated daily intake rate (DIR) values of the consumed PHEs, within the four analyzed groups of fruits, were compared to the tolerable daily intake of trace elements recommended by JECFA (Joint FAO/WHO Expert Committee on Food Additives), World Health Organization (WHO), and The United States Environmental Protection Agency (USEPA) guidelines. Using the provisional maximum tolerable daily intakes (PMTDI), the following values were adopted in the present study (mg/kg bw-day): As 0.0021 [65], Cd 0.0008 [66], Co 0.0014 [67], Cu 0.5 [68], Hg 0.0006 [69], Ni 0.005 [66], Sb 0.006 [70], Tl 0.00014 [71], and Zn 1 [68]. The PHE intake rates, calculated as a percentage of PMTDI (%PMTDI; Figure 6) revealed that, in the case of essential PHEs, the highest Zn and Cu intake values were identified in shell fruit, i.e., walnut, where the mean PHE values of concentration in plants were equal to 1.53% and 0.69% PMTDI, respectively, under the US adult intake scenario. In the group of non-essential elements, the highest intake was found in the cases of Ni (up to 55.2% of PMTDI in pome fruits) and Tl (up to approx. 8.50% of PMTDI in berry and stone fruits). As to the 95th percentile value of the PHE concentration under the US child intake scenario, the highest intake of Cd was also calculated (up to 13.2% of PMTDI at the 95th percentile of PHE concentration in pome fruits under the US adult intake scenario). In the cases of the remaining non-essential PHEs, the intake rates did not exceed several % of PMTDI. As to the mean contents of As in walnuts, the recommended walnut intake of 30 g per day corresponded to the 0.78% of PMTDI, and considering the 95th percentile of the As content, the same corresponded to the 2.50% of PMTDI.

#### 3.3.2. Non-Carcinogenic Risk of PHEs

**Figure 7:**
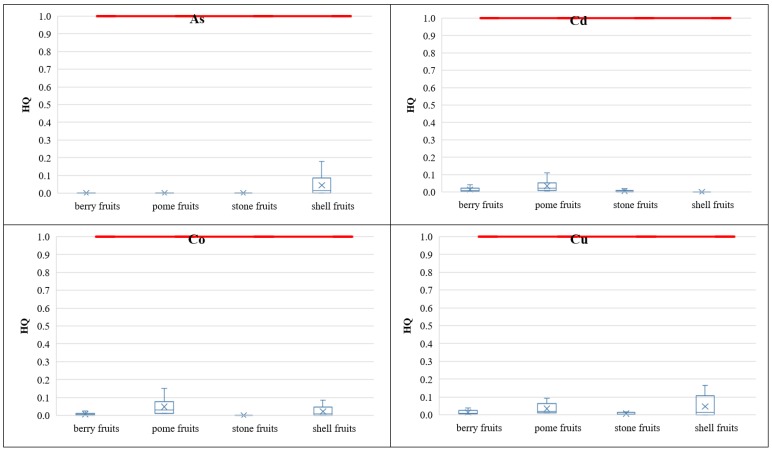

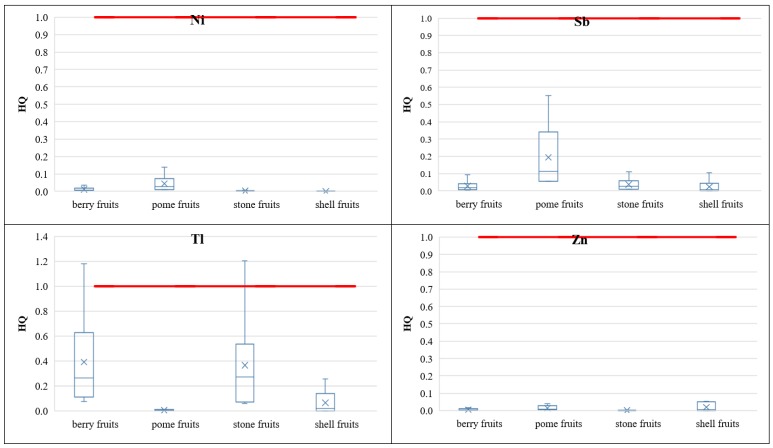

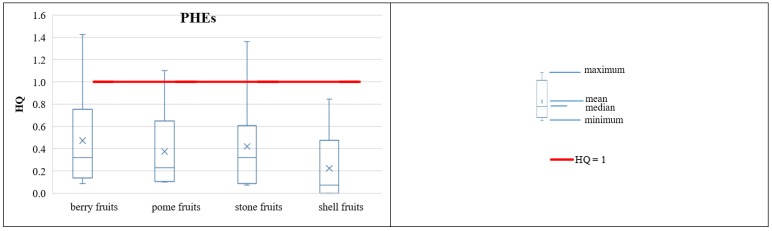
The hazard quotient (HQ) values of PHEs, caused by fruit ingestion under the analyzed fruit intake scenarios.

The target non-carcinogenic risk value described as hazard quotient (HQ) was set to be equal to 1 according to USEPA [7], as well as the Polish Regulation of the Minister of the Environment of 1 September 2016 on the conduct of the assessment of contamination of the surface of the earth [72]. A statistical characterization of the calculated HQ values is presented in Figure 7, involving the respective groups of fruits under the analyzed intake scenarios. For particular PHEs, the target HQ = 1 value was not exceeded, except for the Tl non-carcinogenic risk at the 95th percentile of Tl concentrations in berry (HQ = 1.18) and stone (HQ = 1.20) fruits, under the US child intake scenario. When analyzing fruit groups, the highest mean HQ values were observed in pome fruits: Sb (HQ = 0.19), Co (HQ = 0.05), Ni (HQ = 0.04), and Cd (HQ = 0.03). In each analyzed fruit group the total non-carcinogenic risk did not exceed the target value of 1 as calculated mean risk values were as follows: berry fruits HQ = 0.47, pome fruits HQ = 0.36, stone fruits HQ = 0.42, and shell fruits HQ = 0.22. Considering intake scenarios, the target total non-carcinogenic risk was exceeded at the 95th percentile of PHE contents in the US child intake scenario in following fruit groups: berry fruits (HQ = 1.43), pome fruits (HQ = 1.10), and stone fruits HQ = 1.36.

#### 3.3.3. Carcinogenic Risk of PHEs

**Figure 8:**
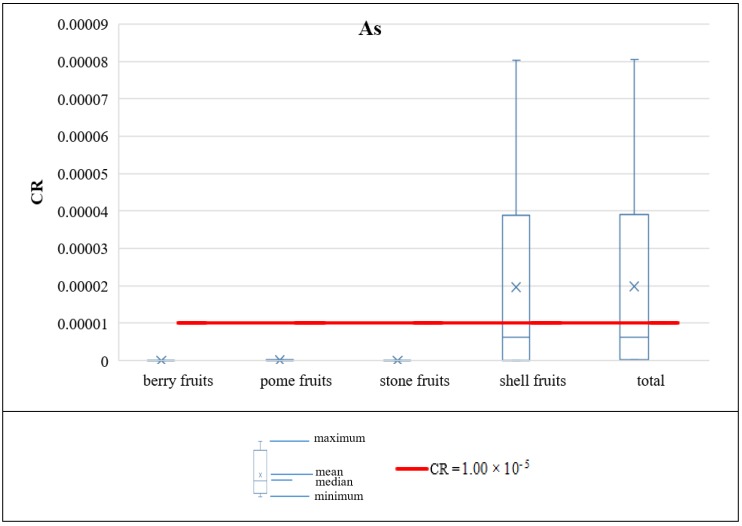
Carcinogenic risk (CR) values for As under the analyzed fruit intake scenarios.

The acceptable carcinogenic risk (CR) level was set to be equal to 1 × 10^−5^, as defined in the Polish Regulation of the Minister of the Environment of 1 September 2016 on the conduct of the assessment of contamination of the surface of the earth [72]. In this study, only As was investigated as potentially carcinogenic, according to the current knowledge of the SF values regarding chemical substances. The statistical characterization of the calculated CR values for the walnut (since As contents were <LOD in other fruit species) is presented in Figure 8 under the analyzed intake scenarios. Under the adult intake scenario, in reference to the Polish statistical consumption rates, the acceptable carcinogenic risk for As was not exceeded for both mean (CR = 1.98 × 10^−6^) and 95th percentile (CR = 6.36 × 10^−6^) for the As content in walnuts. However, the acceptable carcinogenic risk level was exceeded for adults under the recommended 30 g of walnut consumption per day, in respect of both mean (CR = 1.67 × 10^−5^) and 95th percentile (CR = 5.36 × 10^−5^) for the As content in the shell fruit.

#### 3.3.4. Margin of Exposure to Pb

The benchmark dose limit (BMDL) values are used to determine the thresholds below which health risk is considered to be acceptable low [32]. Thus, using the margin of exposure (MOE) approach, the values <1 indicate high health risk, while the values of MOE >1 point at an acceptable low risk. Considering the exposure scenario, with the Polish statistical data for adults and daily fruit consumption, the MOE value was equal to 15.7 for the mean Pb content and the MOE value was equal to 5.52 at the 95th percentile of Pb content (Table 5). Considering the exposure scenario, with the recommended USEPA data for adults and daily fruit consumption, the MOE value was equal to 14.0 for the mean Pb content and the MOE value for Pb content was equal to 4.89 at the 95th percentile. Under the recommended USEPA child intake scenario, the MOE value of the mean Pb content was equal to 1.64. Thus, all the above stated values pointed at an acceptable low risk arising from the Pb content in consumed fruits, collected from the investigated regions of southern Poland. Only in the case of the 95th percentile exposure level for children, according to the USEPA recommended daily intake amount of fruit, the MOE value was equal to 0.58, and it indicated a high health risk at >1.

#### 3.3.5. Uncertainties in HHRA

The small amount of investigated samples for particular fruit samples had an impact on the range as well as on the mean value of investigated PHE contents. The calculated risk values depend strongly on the assumed intake rates. No statistical data were available for children in Poland. The collected statistical data concerned adults and involved only the most popular fruits consumed by people. The attempted HHRA assumed that the investigated fruits constituted 100% of the PHE intake, excluding other ingestion sources, as well as other exposure pathways, i.e., dermal contact or inhalation.

## 4. Conclusions

The presented research project investigated the PHE contents in four groups of fruits (berry, pome, shell, and stone), cultivated precisely on arable soils in the southern Poland. Results of arable soil investigations were described previously in [13,14]. The maximum allowable concentration (MAC) values stated by the European Union regulation were exceeded in investigated samples for Pb in berry, pome, and stone fruits and for Cd in pome fruits. In respect of the bioavailable PHE content in soil (fraction F1 of the BCR three step sequential extraction procedure 0.11 mol/dm^3^ CH_3_COOH), the potential of Cu accumulation was observed in all four groups of fruits, of Sb in pome, stone, and berry fruits, of Ni in pome and berry fruits, and of Tl in stone and berry fruits. The highest intake rates of essential PHEs, as a percentage of the permissible maximum tolerable daily intake (PMTDI), were identified in shell fruit, i.e., walnut, as follows: Zn 1.53% and Cu 0.69% of PMTDI under the US adult intake scenario. In respect of the non-essential elements, the highest intake was stated for Ni (up to 55.2% of PMTDI in pome fruits) and Tl (up to approx. 8.50% of PMTDI in berry and stone fruits). In respect of the remaining non-essential PHEs, the intake rates did not exceed several per cent of PMTDI.

The target non-carcinogenic risk was exceeded only at the 95th percentile of Tl concentrations in berry (HQ = 1.18) and stone (HQ = 1.20) fruits, under the US child intake scenario. The total non-carcinogenic risk posed by particular fruit groups was not exceeded since the mean HQ_total_ values were as follows: berry fruits HQ = 0.47, pome fruits HQ = 0.36, stone fruits HQ = 0.42, and shell fruits HQ = 0.22. Under the assumed intake scenarios, the target total non-carcinogenic risk values were exceeded only under the US child intake scenario at the 95th percentile of PHE contents as follows: berry fruits HQ = 1.43, pome fruits HQ = 1.10, and stone fruits HQ = 1.36. An acceptable carcinogenic risk of As was not exceeded under an adult intake scenario, based on the Polish statistical consumption rates, in respect of both mean (CR = 1.98 × 10^−6^) and 95th percentile (CR = 6.36 × 10^−6^) of the As content in walnuts. The risk from Pb intake via fruit consumption was acceptable low, according to the margin of exposure (MOE) approach. Only in the case of the 95th percentile exposure level by children, in reference to the USEPA recommended daily intake amount of fruit, the MOE value was equal to 0.58, and when being higher than 1, it indicated a high health risk.

## Figures and Tables

**Table 1:** Description of the fruits investigated in the present study.

No.	Common Name	Botanical Name	Number of Samples (*n*)	Group of Fruit
1.	Black currant	*Ribes nigrum*	3	berry fruit
2.	Blackberry	*Rubus fruticosus*	2	berry fruit
3.	Blueberry	*Vaccinium myrtillus*	2	berry fruit
4.	Gooseberry	*Ribes uva-crispa*	4	berry fruit
5.	Grapes	*Vitis vinifera*	5	berry fruit
6.	Raspberry	*Rubus idaeus*	5	berry fruit
7.	Red currant	*Ribes rubrum*	6	berry fruit
8.	Strawberry	*Fragaria × ananassa Duchesne*	6	berry fruit
9.	Wild strawberry	*Fragaria vesca*	2	berry fruit
10.	Apple	*Malus domestica*	7	pome fruit
11.	Chokeberry	*Aronia melanocarpa*	3	pome fruit
12.	Hawthorn	*Crataegus oxyacantha*	1	pome fruit
13.	Pear	*Pyrus communis*	4	pome fruit
14.	Rosehip	*Rosa canina*	3	pome fruit
15.	Walnut	*Juglans regia*	6	shell fruit
16.	Apricot	*Prunus armeniaca*	4	stone fruit
17.	Cherry	*Prunus cerasus*	5	stone fruit
18.	Gean	*Prunus avium*	6	stone fruit
19.	Nectarine	*Prunus persica* var. *nucipersica*	2	stone fruit
20.	Peach	*Prunus persica*	2	stone fruit
21.	Plum	*Prunus domestica*	9	stone fruit

**Table 2:** Intake rates (IR), in g ww./person-day, of the consumed groups of fruits.

IR (g ww./Person-Day)			
Type of Fruit	Adult PL	Adult USEPA	Child USEPA
Berry fruits	14.0	23.8	18.0
Pome fruits	38.4 *	37.1	39.3
Stone fruits	12.3	9.80	10.1
Shell fruits	3.56 **	30.0 ***	nd
Sum	68.3	100.7	67.4

Adult PL—daily intake rate for adults, statistical data for Poland [24]. Adult USEPA—daily intake rate for adults, United States Environmental Protection Agency (USEPA) recommended values [25]. Child USEPA—daily intake rate for children, USEPA recommended values [25]. nd—not determined. * data concerning apple consumption. ** data including nuts, seeds, edible stones and dried fruits. *** recommended value for improving health condition [26,27]. IR: intake rate, ww.: wet weight.

**Table 3:** Concentrations of potentially harmful elements (PHEs; mg/kg ww.) in the investigated groups of fruits.

PHEs	Statistical Parameters	Berry Fruits (*n* = 35)	Pome Fruits (*n* = 18)	Shell Fruits (Walnut) (*n* = 6)	Stone Fruits (*n* = 28)
As	min	ND	ND	nd	ND
	mean			0.039	
	SD			0.013	
	max			0.143	
	P95			0.125	
Cd	min	nd	nd	ND	nd
	mean	0.023	0.021		0.012
	SD	0.006	0.005		0.004
	max	0.081	0.116		0.069
	P95	0.058	0.044		0.027
	MAC	0.05			
Co	min	nd	nd	nd	ND
	mean	0.006	0.010	0.023	
	SD	0.002	0.006	0.006	
	max	0.014	0.058	0.062	
	P95	0.013	0.023	0.060	
Cu	min	0.003	0.006	3.082	nd
	mean	0.706	0.710	8.105	0.797
	SD	0.142	0.092	0.202	0.120
	max	2.300	2.292	15.478	2.754
	P95	1.356	1.254	14.603	1.576
Ni	min	nd	nd	ND	nd
	mean	0.414	0.701		0.186
	SD	0.323	0.390		0.117
	max	1.624	2.230		0.790
	P95	0.967	1.300		0.581
Pb	min	nd	nd	ND	nd
	mean	0.166	0.095		0.203
	SD	0.021	0.008		0.020
	max	0.713	0.390		2.069
	P95	0.322	0.200		0.520
	MAC	0.20/0.10			
Sb	min	nd	nd	nd	nd
	mean	0.016	0.058	0.024	0.029
	SD	0.008	0.016	0.008	0.009
	max	0.077	0.240	0.121	0.197
	P95	0.034	0.328	0.097	0.081
Tl	min	nd	nd	nd	nd
	mean	0.006	0.003	0.002	0.013
	SD	0.001	0.001	0.001	0.001
	max	0.050	0.009	0.007	0.064
	P95	0.020	0.008	0.006	0.051
Zn	min	0.78	0.37	31.9	0.49
	mean	2.31	2.55	35.6	1.54
	SD	0.96	1.07	1.99	0.76
	max	4.76	5.34	37.7	3.75
	P95	3.24	3.14	37.7	2.78

nd < limit of detection (LOD); ND < LOD in all samples; P95—the 95th percentile; MAC—maximum allowable concentration; SD—standard deviation.

**Table 4:** Factor loading results obtained from the principal component analysis (PCA) for fruits samples.

PHEs	Varimax Rotated			
	PC1	PC2	PC3	PC4
Cd	−0.008	**−0.871**	−0.119	−0.115
Co	**0.886**	−0.347	0.116	0.149
Cu	**0.958**	0.132	−0.006	−0.221
Ni	0.131	**−0.866**	0.015	0.397
Pb	−0.232	−0.372	−0.219	**−0.837**
Sb	0.039	0.411	**−0.744**	0.192
Tl	−0.148	0.208	**0.835**	−0.094
Zn	**0.939**	0.231	0.002	−0.201
Eigenvalues	2.68	2.05	1.33	1.03
Explained variance %	33.5	25.6	16.6	12.9
Cumulative variance %	33.5	59.1	75.7	88.5

Factor loadings exceeding 0.7 are shown in **bold**. PC1: principal component 1, PC2: principal component 2, PC3: principal component 3, PC4: principal component 4.

**Table 5:** Margin of exposure (MOE) values for Pb under the analyzed fruit intake scenarios.

Fruit Intake Scenario	MOE (Mean Exposure)	MOE (P95 Exposure)
Adult PL	15.7	5.52
Adult USEPA	14.0	4.89
Child USEPA	1.64	0.58

P95—the 95th percentile. Adult PL—intake rate for adults, statistical data for Poland [24]. Adult USEPA—daily intake rate for adults, United States Environmental Protection Agency (USEPA) recommended values [25]. Child USEPA—daily intake rate for children, USEPA recommended values [25].

## Supplementary Materials

The following are available online at https://www.mdpi.com/1660-4601/16/24/5096/s1, Table S1. Measuring parameters of ICP spectrometers used in the study, Table S2. Results of one-way ANOVA of differences between average concentrations of PHEs in groups of fruits, Table S3. Results of one-way ANOVA of differences between average concentrations of PHEs in investigated regions of southern Poland, Table S4. Selected PHE contents in arable soils in southern Poland (based on [13,14]) used for soil-to-plant transfer indices in this study.
