# Supplementary material for: Human Health Risk Assessment and Potentially Harmful Element Contents in the Fruits Cultivated in the Southern Poland
Source: Int J Environ Res Public Health. 2019 Dec 13;16(24):5096. doi: 10.3390/ijerph16245096 (PMC6950181; doi:10.3390/ijerph16245096)
Supplement: Supplementary file 1 [file ijerph-16-05096-s001.pdf]

International Journal of
Environmental Research and Public Health

MDPI

Supplementary material

Human health Risk Assessment and Potentially Harmful Element Contents in the Fruits Cultivated in the Southern Poland

Agnieszka Gruszecka-Kosowska

Department of Environmental Protection, Faculty of Geology, Geophysics, and Environmental Protection, AGH University of Science and Technology, Al. Mickiewicza 30, 30-059 Kraków, Poland; agnieszka.gruszecka@agh.edu.pl

Received: 10 November 2019; Accepted: 09 December 2019; Published: date

Table S1. Measuring parameters of ICP spectrometers used in the study.

Table S2. Results of one-way ANOVA of differences between average concentrations of PHEs in groups of fruits.

Table S3. Results of one-way ANOVA of differences between average concentrations of PHEs in investigated regions of southern Poland.

Table S4. Selected PHE contents in arable soils in southern Poland (based on [1,2]) used for soil-to-plant transfer indices in this study.

Table S1. Measuring parameters of ICP spectrometers used in the study.

Sample Introduction Compartment/Parameter	ELAN 6100 Inductively Coupled Plasma Mass Spectrometer (ICP-MS)	Optima 7300DV Inductively Coupled Plasma Optical Emission Spectrometer (ICP-OES)
Torch	Standard alumina injector with a 2.0 mm inner diameter	Quartz
Spray Chamber	Double-pass Scott-type	Double-pass Scott-type
Nebulizer	The Gem Tip Cross-Flow, pneumatic	Cross-Flow
Radio Frequency	40 MHz	40.16 MHz
RF Generator	1300 W	1050 W
Plasma Flow	15 L/min	15 L/min
Auxiliary Flow	0.2 L/min	1.5 L/min
Nebulizer Flow	0.8 L/min	0.93 L/min
Sample Flow Rate	1.5 mL/min	1.5 mL/min
Equilibration Time	30 s	400–3000 ms
Wavelength	Zn—206.200 nm	na
Monitored Isotopes	na	^{75}As, ^{114}Cd, ^{59}Co, ^{53}Cr, ^{63}Cu, ^{202}Hg, ^{58}Ni, ^{208}Pb, ^{121}Sb, ^{82}Se, ^{205}Tl
Internal Standard	na	^{89}Y
Plasma View	Axial	na
Replicates	3	3

RF—radio frequency, na—not applicable

Table S2. Results of one-way ANOVA of differences between average concentrations of PHEs in groups of fruits.

PHEs	F	*p*	Confidence Interval	Fisher’s LSD Test, Probabilities for Post-Hoc Tests				
Cd	0.7625	0.5197	0.95	Non-significant differences				
Co	5.6909	**0.0017**	0.95	Error: between MS = 0.00010, df = 58.000				
					{1}	{2}	{3}	{4}
					0.0016	0.0005	0.0066	0.0219
				berry fruits		0.7160	0.1710	**0.0004**
				stone fruits	0.7160		0.1176	**0.0003**
				pome fruits	0.1710	0.1176		**0.0115**
				shell fruits	**0.0004**	**0.0003**	**0.0115**	
Cu	61.128	**0.00000**	0.95	Error: between MS = 1.0285, df = 58.000				
					{1}	{2}	{3}	{4}
					0.6152	0.8301	0.8193	7.8118
				berry fruits		0.4787	0.5738	**0.0000**
				stone fruits	0.4787		0.9777	**0.0000**
				pome fruits	0.5738	0.9777		**0.0000**
				shell fruits	**0.0000**	**0.0000**	**0.0000**	
Ni	2.1383	0.1052	0.95	Non-significant differences				
Pb	0.8197	0.4883	0.95	Non-significant differences				
Sb	1.1131	0.3512	0.95	Non-significant differences				
Tl	0.1694	0.9166	0.95	Non-significant differences				
Zn	874.99	**0.00000**	0.95	Error: between MS = 1.5541, df = 58.000				
					{1}	{2}	{3}	{4}
					2.5368	1.5179	2.4569	35.155
				berry fruits		**0.0079**	0.8577	**0.0000**
				stone fruits	**0.0079**		0.0515	**0.0000**
				pome fruits	0.8577	0.0515		**0.0000**
				shell fruits	**0.0000**	**0.0000**	**0.0000**	

PHEs—potentially harmful elements, F—F-ratio, *p*—probability, values <0.05 are shown in bold.

Table S3. Results of one-way ANOVA of differences between average concentrations of PHEs in investigated regions of southern Poland.

PHEs	F	*p*	Confidence Interval	Fisher's LSD Test, Probabilities for Post-Hoc Tests				
Cd	2.5568	0.0639	0.95	Non-significant differences				
Co	2.9642	**0.0394**	0.95	Error: between MS = 0.00011, df = 58.000				
					{1}	{2}	{3}	{4}
					0.00015	0.00005	0.00961	0.00703
				Opolskie		0.9771	**0.0228**	0.0672
				Śląskie	0.9771		**0.0241**	0.0700
				Małopolskie	**0.0228**	**0.0241**		0.5444
				Świętokrzyskie	0.0672	0.0700	0.5444	
Cu	1.2622	0.2958	0.95	Non-significant differences				
Ni	2.3595	0.0808	0.95	Non-significant differences				
Pb	0.6396	0.5926	0.95	Non-significant differences				
Sb	3.6244	**0.0181**	0.95	Error: between MS = 0.00204, df = 58.000				
					{1}	{2}	{3}	{4}
					0.0423	0.0042	0.0359	0.0005
				Opolskie		**0.0142**	0.7077	**0.0095**
				Śląskie	**0.0142**		0.0750	0.8179
				Małopolskie	0.7077	0.0750		0.0532
				Świętokrzyskie	**0.0095**	0.8179	0.0532	
Tl	1.8459	0.1489	0.95	Non-significant differences				
Zn	0.0320	0.9922	0.95	Non-significant differences				

PHEs—potentially harmful elements, F—F-ratio, *p*—probability, values <0.05 are shown in bold.

Table S4. Selected PHE contents in arable soils in southern Poland (based on [1,2]) used for soil-to-plant transfer indices in this study.

PHEs	Mean total content of PHE in arable soils from southern Poland [1]	Mean content of PHEs in exchangeable and acid soluble forms in arable soils from southern Poland [2]	Mean potential soluble total PHE concentration in pore water of arable soils from southern Poland [2]
	extraction with aqua regia	extraction with 0.11 mol/dm^3 CH_3COOH in first step of the BCR sequential extraction procedure	extraction with 0.05 mol/dm^3 Na_2EDTA
		mg/kg dw.	
As	6.64	0.28	na
Cd	0.39	0.45	0.72
Co	4.92	0.44	na
Cu	26.6	0.72	4.45
Ni	11.5	0.64	1.29
Pb	63.8	<LOD	21.3
Sb	1.23	0.04	na
Tl	0.1	0.005	na
Zn	283	63.6	40.3

PHEs—potentially harmful elements, BCR—Community Bureau of Reference, dw.—dry weight.

References

1. Gruszecka-Kosowska, A.; Baran, A.; Wdowin, M.; Mazur-Kajta, K.; Czech, T. The contents of the Potentially Harmful Elements in the arable soils of southern Poland, with the assessment of ecological and health risks: A case study. *Environ. Geochem. Health* **2019**, doi.org/10.1007/s10653-019-00372-w.
2. Gruszecka-Kosowska, A.; Baran, A.; Mazur-Kajta, K.; Czech, T. Geochemical fractions of the agricultural soils of southern Poland and the assessment of the Potentially Harmful Element mobility. *Minerals* **2019**, *9*, 674, doi:10.3390/min9110674.

© 2019 by the authors. Submitted for possible open access publication under the terms and conditions of the Creative Commons Attribution (CC BY) license (http://creativecommons.org/licenses/by/4.0/).
